# Assessment of T2-Weighted Coronal Magnetic Resonance Images in the Investigation of Pituitary Lesions

**DOI:** 10.1155/2014/650926

**Published:** 2014-03-23

**Authors:** Ruken Yuksekkaya, Levent Aggunlu, Yusuf Oner, Halil Celik, Sergin Akpek, Fatih Celikyay

**Affiliations:** ^1^Radiology Department, School of Medicine, Gaziosmanpasa University, 60100 Tokat, Turkey; ^2^Radiology Department, Afyonkarahisar State Hospital, 03300 Afyonkarahisar, Turkey; ^3^Radiology Department, School of Medicine, Gazi University, 06170 Besevler, Ankara, Turkey; ^4^Radiology Department, Kahramanmaras Megapark Hospital, 46000 Kahramanmaras, Turkey; ^5^Radiology Department, American Hospital, 34365 Istanbul, Turkey

## Abstract

Magnetic resonance imaging is the most important diagnostic method in the investigation of the pituitary lesions. Our aim is to determine whether T2-weighted coronal images may be helpful in the evaluation of the pituitary gland with suspected pituitary adenomas. One hundred and sixty-seven patients were examined prospectively with T2-weighted coronal and T1-weighted coronal images enhanced with intravenous contrast material. The images were evaluated for the presence, the size, the location, and the ancillary signs including sellar floor erosion or ballooning, infindibulary deviation, convexity of the superior border of the gland, diffuse enlargement of the gland, and the invasion of the cavenous sinuses on both images. In forty-six (28%) patients lesions were revealed on both sequences. In twenty-one (12%) patients the lesions that were revealed on the T1-weighted images were not detected on the T2-weighted images. Positive predictive value, negative predictive value, sensitivity, specificity, and diagnostic accuracy rates of T2-weighted coronal images on the detection of the presence of lesions were 100%, 17.4%, 68.7%, 100%, and 87.4%, respectively. Both T2-weighted coronal and T1-weighted coronal images enhanced with intravenous gadolinium-based contrast material are important in the diagnosis of pituitary adenomas. T2-weighted coronal images could be used as a screening tool for the primary evaluation of the pituitary gland.

## 1. Introduction

Pituitary adenomas are the most common pathologies encountered in the sellar area [1]. They also either may be clinically silent or can cause a variety of symptoms related to compression of adjacent structures and hormone secretion. Today, magnetic resonance (MR) imaging is usually the first diagnostic method in the investigation of pituitary adenomas. The detection of adenomas on MR imaging depends on the image contrast between adenoma and normal pituitary tissue. There are also ancillary signs including sellar floor erosion or ballooning, infindibulary deviation, convexity of the superior border of the gland, diffuse enlargement of the gland, and the invasion of the cavernous sinuses [2]. T1-weighted coronal images enhanced with intravenous gadolinium-based contrast material (IV-GBCM) provide higher detection rates of adenomas. T2-weighted coronal images may be important for the detection of the pituitary adenomas without administration of IV-GBCM. Therefore, time and account of the examinations will be decreased and the cost effectiveness will be increased. Also possible harmful side effects of the IV-GBCM such as nephrogenic systemic fibrosis (NSF) will be prevented. We aimed to determine whether the T2-weighted coronal images may be helpful in the evaluation of the pituitary gland with MR imaging in a large amount of patients that clinically and laboratorily suspected pituitary adenomas.

## 2. Material and Methods

A total of 167 patients (125 women, 42 men; age range 7–67,mean age 35 ± 14) who underwent MR imaging because of symptoms and laboratory signs of the pituitary dysfunction were included in this study. This study was approved by the Institutional Review Board and informed consent from each subject was obtained.

Patients were examined on a 1.5 Tesla superconducting MR system (Signa Excite II, GE Healthcare Milwaukee, WI, USA). The imaging protocol included T2-weighted axial fast spin-echo (FSE) sequence (TR/TE, 4600/85 ms; matrix, 320-224; field of view, 24 cm; section thickness, 5 mm; intersection gap, 2 mm; NEX, 2), T2-weighted coronal FSE sequence (TR/TE, 3200/102 ms; field of view, 18 cm; section thickness, 3 mm; intersection gap, 0.2 mm; matrix, 320-224; NEX, 4), and T1-weighted coronal and sagittal spin-echo (SE) sequence (TR/TE, 500 ms/min full; field of view, 18 cm; section thickness, 3 mm; intersection gap, 0.2 mm; matrix, 320-224; NEX: 4) before and after administration of IV-GBCM. The scanning time of T1-weighted coronal sequences with IV-GBCM is 2.25 minutes and T2-weighted coronal sequences with IV-GBCM is 2.10 minutes.

Three observers reviewed each image and reached a consensus without knowledge of the clinical findings. T1-weighted coronal images enhanced with IV-GBCM were considered gold standard for the evaluation of the pituitary gland. For all examinations, the presence, the size, the location, and the ancillary signs of the focal lesions were evaluated on the T1-weighted coronal images enhanced with IV-GBCM and T2-weighted coronal images. A focal lesion was considered present when a focus of signal alteration was separable from the normal appearing pituitary tissue. Lesion size was measured on both images. Lesions were classified as microadenomas (≤10 mm) or macroadenomas (>10 mm) according to their size. Location of the lesions was charted as right, left, middle of the gland, and diffusely enlarged gland. Also all patients were evaluated carefully for the ancillary signs on the both images. Ancillary signs include sellar floor erosion or ballooning, infindibulary deviation, convexity of the superior border of the gland, diffuse enlargement of the gland, and the invasion of the cavernous sinuses.

Continuous data was expressed as the mean ± standard deviation (SD) and categorical data as numbers with related percentages (*n*, %). Differences in continuous data were analyzed by using Student's *t*-test. A two-tailed *P* value < 0.05 was considered to be statistically significant. All statistical analyses were performed using the Statistical Package for the Social Sciences software package version 15.0 (SPSS Inc., Chicago, Illinois, USA). The MR signs previously mentioned were compared between the T1-weighted coronal images enhanced with IV-GBCM and T2-weighted coronal images on the basis of the clinical history and hormone assay. Positive predictive value (PPV), negative predictive value (NPV), sensitivity, specificity, and diagnostic accuracy rates were estimated.

## 3. Results

**Figure 1:**
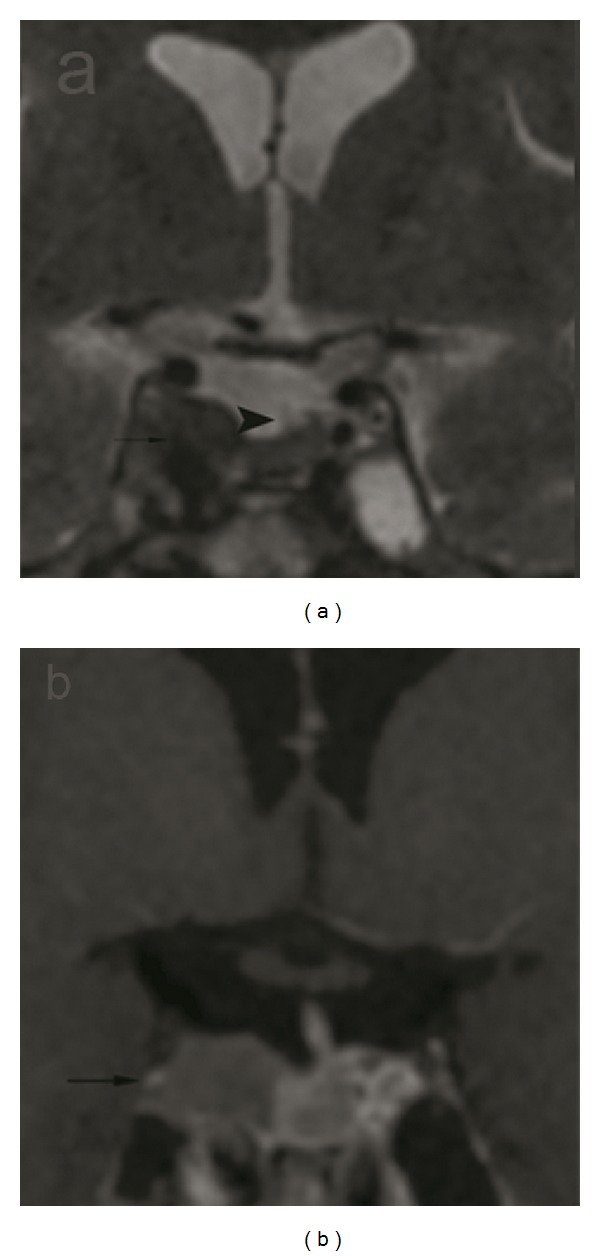
A hyperintense macroadenoma with the infindibulary deviation (arrow heads) and the invasion of the right cavernous sinus (arrows) are shown on the T2-weighted coronal image (a) and T1-weighted coronal image enhanced with IV-GBCM (b) of a patient.

**Figure 2:**
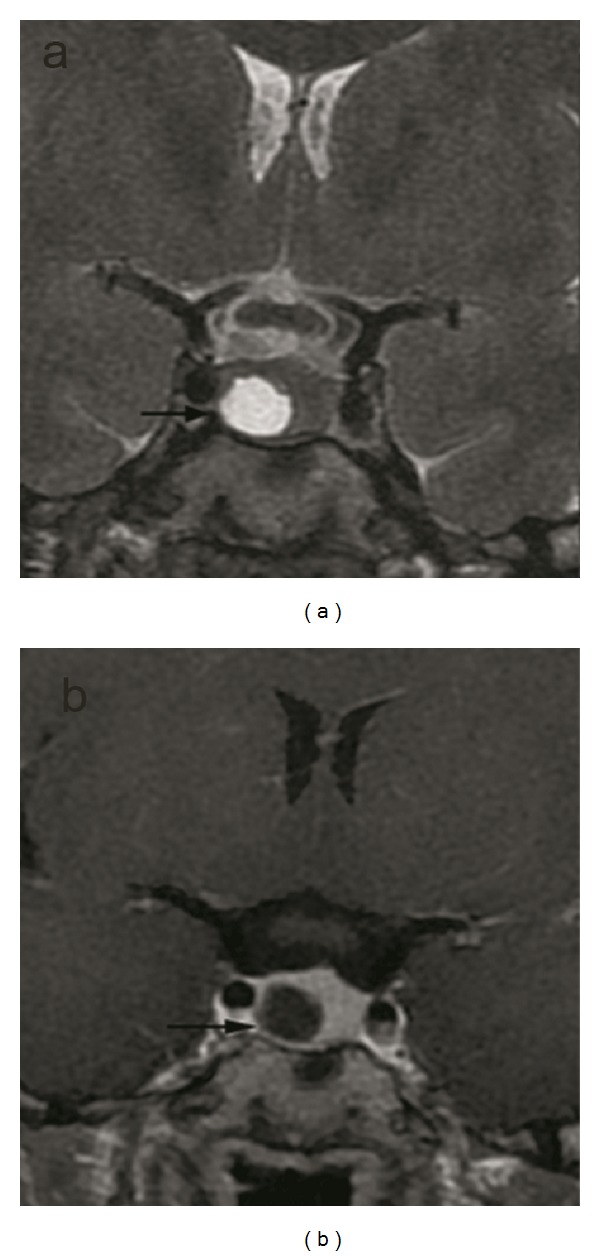
Rathke's cleft cyst or cystic macroadenoma with hyperintense signal intensity on the T2-weighted coronal image (a) (arrows) and hyperintense signal intensity on the T1-weighted coronal image enhanced with IV-GBCM (b) (arrows) are shown. Diffuse enlargement of the gland and the invasion of the right cavernous sinus can be demonstrated on both images.

**Figure 3:**
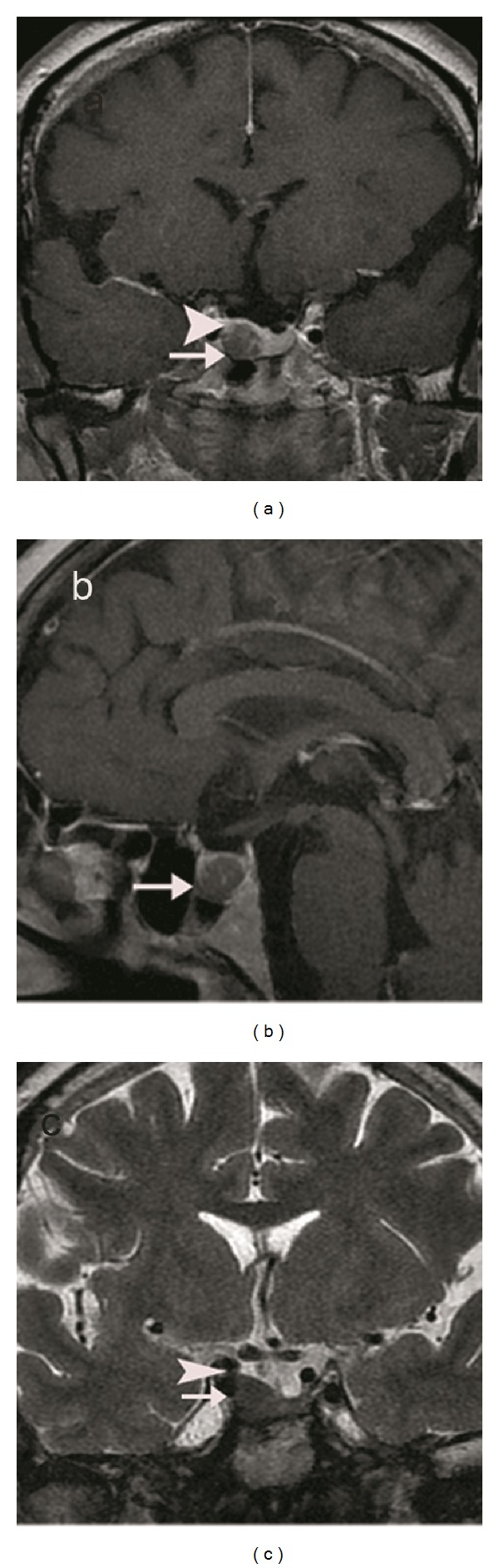
T1-weighted coronal (a) and sagittal (b) images enhanced with IV-GBCM and T2-weighted coronal (c) images are showing a hyperintense macroadenoma. Convexity of the right side of the gland (arrow heads) and the invasion of the right cavernous sinus (arrows) can be demonstrated on both T1-weighted and T2-weighted images.

**Figure 4:**
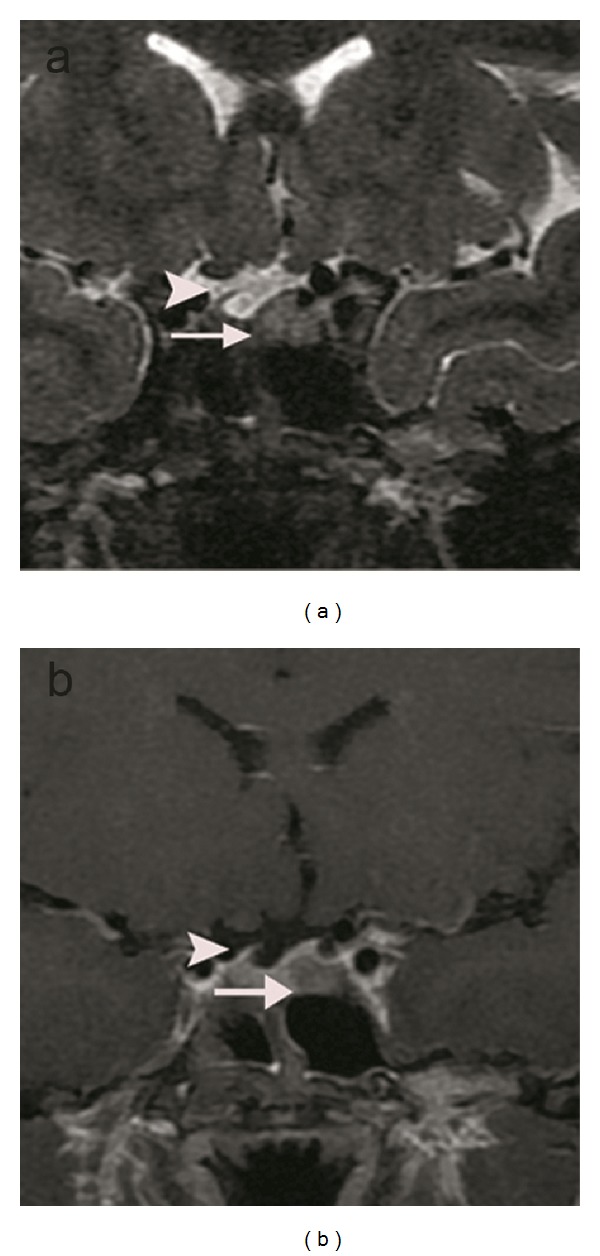
T2-weighted coronal image (a) shows a suspicious hyperintense lesion on the left side of the gland. T1-weighted coronal image enhanced with IV-GBCM (b) shows the lesion as a hyperintense lesion on the left side of the gland. Infindibulary deviation (arrow heads) and diffuse enlargement of the pituitary gland can be observed on both images.

**Figure 5:**
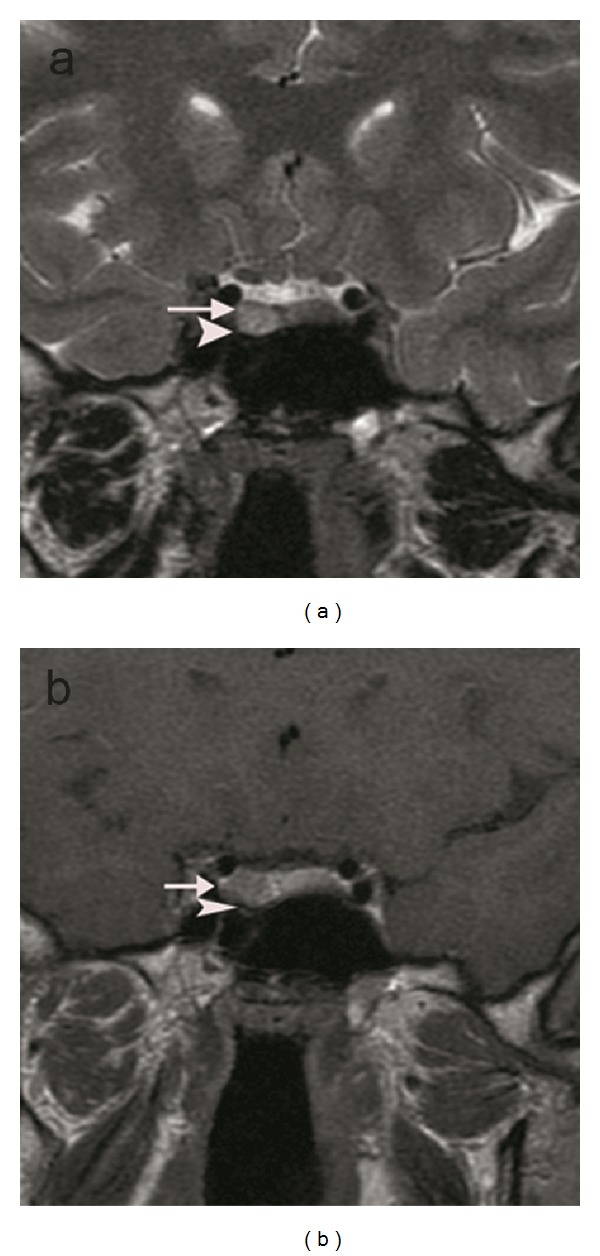
A microadenoma with hyperintensity on T2-weighted coronal (a) and hyperintensity on the T1-weighted coronal image enhanced with IV-GBCM (b) (arrows) on the right side of the gland is shown. Sellar floor ballooning (arrow heads) can be seen on both images.

**Figure 6:**
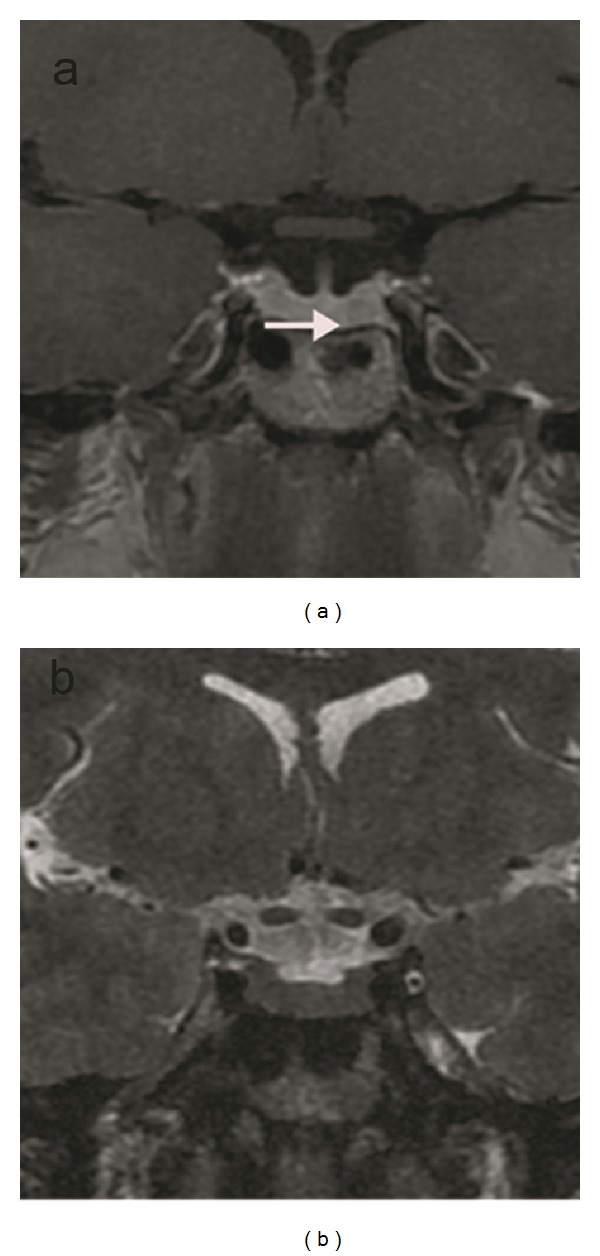
A microadenoma with hyperintensity on the T1-weighted coronal image enhanced with IV-GBCM on left side of the gland is shown (a) (arrow). T2-weighted coronal image (b) shows no abnormalities.

In 46 (27.5%) patients, lesions were revealed on the T2-weighted coronal images. In 67 (40.1%) patients, lesions were revealed on the T1-weighted coronal images enhanced with IV-GBCM. All of the lesions identified on the T2-weighted coronal images were also identified on the T1-weighted coronal images enhanced with IV-GBCM (Figures 1, 2, 3, 4, and 5). The lesions of the 21 patients (11.9%) that were revealed on the T1-weighted coronal images enhanced with IV-GBCM were not detected on the T2-weighted coronal images (Figure 6). The patients that were evaluated as being normal on the T2-weighted coronal images were also normal on the T1-weighted coronal images enhanced with IV-GBCM. When we considered the T1-weighted coronal images enhanced with IV-GBCM as gold standard, PPV, NPV, sensitivity, specificity, and diagnostic accuracy rates of the T2-weighted coronal images in the detection of the presence of lesions were 100%, 17.4%, 68.7%, 100%, and 87.4%, respectively. There was no false positive case on the T2-weighted coronal images.

All of the lesions that were detected as microadenoma on T1-weighted coronal images enhanced with IV-GBCM were detected also as microadenoma on T2-weighted coronal images. Three lesions that were detected as macroadenoma on T1-weighted coronal images enhanced with IV-GBCM (15%) were defined as microadenoma on T2-weighted coronal images.

The locations of the lesions were the same at all patients on both images (Figures 1–5). When T1-weighted coronal images enhanced with IV-GBCM were considered gold standard images, sensitivity and specificity of T2-weighted coronal images on ancillary MR imaging signs were such as 92–100% at infindibulary deviation, 89–100% at convexity of the superior border of gland, 94–100% at diffuse enlargement of the gland, 92–98% at sellar floor destruction, and 92–99% at the invasion of the cavernous sinuses, respectively (Figures 1–6). The PPV rates of T2-weighted coronal images were such as 100% at infindibulary deviation, convexity of the superior border of gland, and diffuse enlargement of the gland; 81% at sellar floor destruction; and 92% at the invasion of the cavernous sinuses. The sensitivity, specificity, and PPV rates of T2-weighted coronal images in the evaluation of the presence of any of an ancillary findings were 98%, 90.7%, and 99%, respectively.

## 4. Discussion

In the planning of treatment options for the pituitary adenomas, there is a demand for accurate knowledge of the presence and extension of the lesion. Various imaging techniques have been described for imaging of pituitary lesions. Computed tomography with high resolution techniques has been a method of imaging in the diagnosis of pituitary abnormalities [3]. Currently, MR imaging is an important method for the morphological investigation of the pituitary adenomas. Soft tissue contrast, absence of the beam hardening artefacts due to adjacent bone, good spatial resolution, and detection of the gland in multiplanar images were considered extremely important advantages of this modality [4]. While many imaging techniques have been experimented, the most well-known conventional sequences are thin-section T1-weighted coronal and sagittal SE images before and after administration of contrast material [2, 5, 6]. It is known that T1-weighted images show anatomic details perfectly. Lesions seen typically hyperintense on T1-weighted images enhanced with IV-GBCM. The T1-weighted images enhanced with IV-GBCM may be negative if the tumor is extremely small, the dose of gadolinium is too high, or the visualization window is too large [1]. Also loss of lesion visibility has been noted on delayed contrast enhanced MR imaging studies [7]. It has been suggested that a delay in timing could lead to equalization in gland-lesion contrast and reduction in adenoma detection [8]. On the other hand, Bartynski and Lin [8] noted that in 35% of cases adenomas enhance before the gland.

Also it is known that the administration of contrast material can also cause NSF and renal failure. Nephrogenic systemic fibrosis is a rare multisystemic fibrosing disorder that principally affects the skin but may affect other organs such as heart, lungs, diaphragm, liver, and kidneys, resulting in variable end organ damage and even death of patients with renal insufficiency and decreased kidney function, particularly in the presence of a proinflammatory process such as major surgery, infection, or a vascular thromboembolic event [9]. It is important to avoid the administration of contrast material especially in patients at risk. Also the costs of the contrast material also hold a lot.

We hypothesized that the T2-weighted coronal images may be valuable for screening of the pituitary gland and the T1-weighted coronal images enhanced with IV-GBCM may be more able to confirm and characterise any abnormalities. The T2-weighted coronal images can be a guide. By this method, when the lesion was detected on T2-weighted coronal images, the examination would be ended without administration of IV-GBCM. Thus, time and the cost of the examination will be decreased, the cost effectiveness will be increased, and the harmful effects of contrast material such as NSF will be decreased.

In our series we found 68.7% sensitivity of the T2-weighted coronal images in detection of adenomas. The T2-weighted coronal images showed excellent specificity with no false-positive cases. Stadnik et al. [10] noted 33% sensitivity of the T2-weighted images in detection of adenomas. On the other hand, Kulkarni et al. [3] suggested that they had detected 15 of 17 microadenomas on T2-weighted images, although, they reported that some microadenomas were better seen on T2-weighted images [3]. We demonstrated that the T2-weighted coronal images have high specificity (100%) and low sensitivity (68.7%) rates, in such a way that, when the lesion was observed on the T2-weighted coronal images, the examination would be ended without administration of IV-GBCM.

Although it was believed that the T1-weighted images showed anatomic detail perfectly, on T2-weighted coronal images, the location of the lesions was the same with T1-weighted coronal images enhanced with IV-GBCM. Also only on three cases the estimated sizes of lesions as micro- and macroadenomas between those two images were different. Sensitivity, specificity, and PPV rates of T2-weighted coronal images on ancillary MR imaging signs were excellent.

## 5. Conclusions

The T2-weighted coronal images provide improved visibility of adenomas and ancillary MR imaging signs. They are easy-to-perform and do not need the usage of IV-GBCM. The practice of examining the gland at the beginning of the investigation with T2-weighted coronal images will be effective. In this context, the T2-weighted coronal images are valuable diagnostic images for the primary evaluation of the pituitary gland.
